# Comparison of Health Risk Assessments of Heavy Metals and As in Sewage Sludge from Wastewater Treatment Plants (WWTPs) for Adults and Children in the Urban District of Taiyuan, China

**DOI:** 10.3390/ijerph14101194

**Published:** 2017-10-08

**Authors:** Baoling Duan, Wuping Zhang, Haixia Zheng, Chunyan Wu, Qiang Zhang, Yushan Bu

**Affiliations:** 1 College of Resources and Environment, Shanxi Agricultural University, Taigu 030801, Shanxi, China; sxnddbl@163.com (B.D.); zwping@126.com (W.Z.); zhenghaixia81@126.com (H.Z.); chunyan_wu224@163.com (C.W.); 18334759065@163.com (Q.Z.); 2 College of Forestry, Shanxi Agricultural University, Taigu 030801, Shanxi, China

**Keywords:** heavy metals, human health risk, exposure assessment, non-carcinogenic risk, carcinogenic risk, uncertainty analysis, urban district of Taiyuan

## Abstract

To compare the human health risk of heavy metals and As in sewage sludge between adults and children, samples were collected from five wastewater treatment plants (WWTPs) located in the urban district of Taiyuan, the capital of Shanxi. Heavy metals and As in sewage sludge can be ranked according to the mean concentration in the following order: Cu > Cr > Zn > Pb > As > Hg > Cd. Compared with the concentration limit set by different countries, the heavy metals contents in sewage sludge were all within the standard limits, except for the content of As, which was higher than the threshold limit established by Canada. A health risk assessment recommended by the United States Environmental Protection Agency (USEPA) was used to compare the non-cancer risk and cancer risk between adults and children. Based on the mean and 95% upper confidence limit (UCL) of the average daily dose (ADD), heavy metals and As can be ranked in the order of Cu > Cr > Zn > Pb > As > Hg > Cd for adults, and Cu > Cr > Zn > Pb > Hg > As > Cd for children. Moreover, results of ADD_ingest_ and ADD_inhale_ indicated that ingestion was the main pathway for heavy metals and As exposure for both adults and children, and the sum of ADD implied that the exposure to all heavy metals and As for children was 8.65 and 9.93 times higher, respectively, than that for adults according to the mean and 95% UCL. For the non-carcinogenic risk, according to the hazard quotient (HQ), the risk of Cu, Hg and Cr was higher than the risk of Zn and Pb. The hazard index (HI) for adults was 0.144 and 0.208 for the mean and 95% UCL, which was less than the limit value of 1; for children, the HI was 1.26 and 2.25, which is higher than the limit value of 1. This result indicated that children had non-carcinogenic risk, but adults did not. Furthermore, ingestion was the main pathway for non-carcinogenic risk exposure by the HQ_ingest_ and HQ_inhale_. For the carcinogenic risk, Cd and As were classified as carcinogenic pollutants. The values of RISK for the mean and 95% UCL for adults and children all exceeded the limit value of 1 × 10^−5^, which implied that adults and children had a carcinogenic risk, and this risk was higher for children than for adults. The results of RISK for As and Cd implied that As was the main pollutant for carcinogenic risk. Moreover, the results of RISK_ingest_ and RISK_inhale_ indicated that ingestion was the main pathway. Uncertainty analysis was performed, and the risk ranges of it were greater than certainty analysis, which implied that uncertainty analysis was more conservative than certainty analysis. A comparison of the non-carcinogenic risk and carcinogenic risk for adults and children indicated that children were more sensitive and vulnerable than adults when exposed to the same pollutant in the environment.

## 1. Introduction

Sewage sludge, which is generated during the process of treating municipal wastewater, is quickly increasing [1,2,3]. After the prohibition of ocean disposal of sewage sludge in the 1990s in many countries, the use of sludge as soil amendment or for land reclamation has increased in order to reduce the volume of sludge that must be filled, incinerated, or disposed of at surface sites [4,5,6].

Because sewage sludge contains substances of agricultural value—such as organic matter, nitrogen, phosphorus and potassium—it is regarded as an important resource and can be safely used to condition soils and provide nutrients for agricultural, horticultural, and forest crops and vegetation, as well as for reclaiming and revegetating areas disturbed by mining, construction, and waste disposal activities [4,7,8,9,10]. However, sewage sludge also contains toxic elements and compounds, such as heavy metals, organic toxic compounds and pathogens [11,12,13]. Land spreading involves the transfer of heavy metals from sewage sludge to the soil, and then to the air and water [14]. Because of their bioaccumulation, persistence and toxicity, heavy metals cannot be disintegrated by physical processes and they remain in the environment for a long time. Excessive accumulation of heavy metals in the environment not only poses potential hazards to ecological systems but also, depending on their proximity to human activities, may increase human health exposure to heavy metals and directly affect human health [15,16,17]. 

Heavy metals in sewage sludge can be transferred to the human body, where they accumulate in human fatty tissues, and subsequently affect the nervous system, endocrine system, immune system, hematopoietic function and normal cellular metabolism, etc. [16,18]. To protect public health from the reasonably anticipated adverse effects of heavy metals potentially present in sewage sludge, the U.S. Environmental Protection Agency (USEPA) has developed a comprehensive, risk-based rule commonly known as the Part 503 rule [19]. The regulation (40 CFR Part 503) was published in the Federal Register on 19 February 1993. The rule underwent an extensive multi-pathway risk assessment for evaluating and setting limits to manage heavy metals in sewage sludge. Additionally, an extensive health risk assessment was conducted based on the best scientific data available, established risk guidelines, and the scientific judgment of experts, regarding the land use of sewage sludge [20,21]. The health risk assessment procedure of the USEPA is followed in the present study, and uncertainty analysis was also performed to ensure the accuracy.

Most health risk assessments associated with human exposure to heavy metals in soil, water and air are based on the exposure models formulated by the USEPA [19]. However, there are fewer health risk assessments that focus on children than on adults. Because children have a low tolerance to toxins as well as the inadvertent behavior of coming into contact with significant quantities of sewage sludge, the heavy metals exposure risk for both adults and children should be assessed [4,22,23]. In this study, in order to evaluate the different tolerance levels of adults and children when they are exposed to the same contents of heavy metals in the environment, a comparison of the non-carcinogenic and carcinogenic risks caused by heavy metals and As in sewage sludge between adults and children was performed.

Taiyuan, an important heavy industrial area of China, has an area of 6999 km^2^ and a population of 4.29 million [24]. Heavy industries—such as metallurgy and coking—located in the city, produce local pollution of Cu, Zn, Hg, Pb, Cd and Cr, while the busy traffic produces Pb, Cu and Zn contamination. These heavy metal emissions pose a threat to the health of local residents, especially to children.

The aims of the present study were (a) to determine the concentration of heavy metals in sewage sludge from five wastewater treatment plants (WWTPs) distributed in Taiyuan; (b) to evaluate the exposure of adults and children to heavy metals in sewage sludge; (3) to compare the non-carcinogenic risk between both adults and children; and (4) to compare the carcinogenic risk between both adults and children.

## 2. Materials and Methods 

### 2.1. Sampling

**Figure 1:**
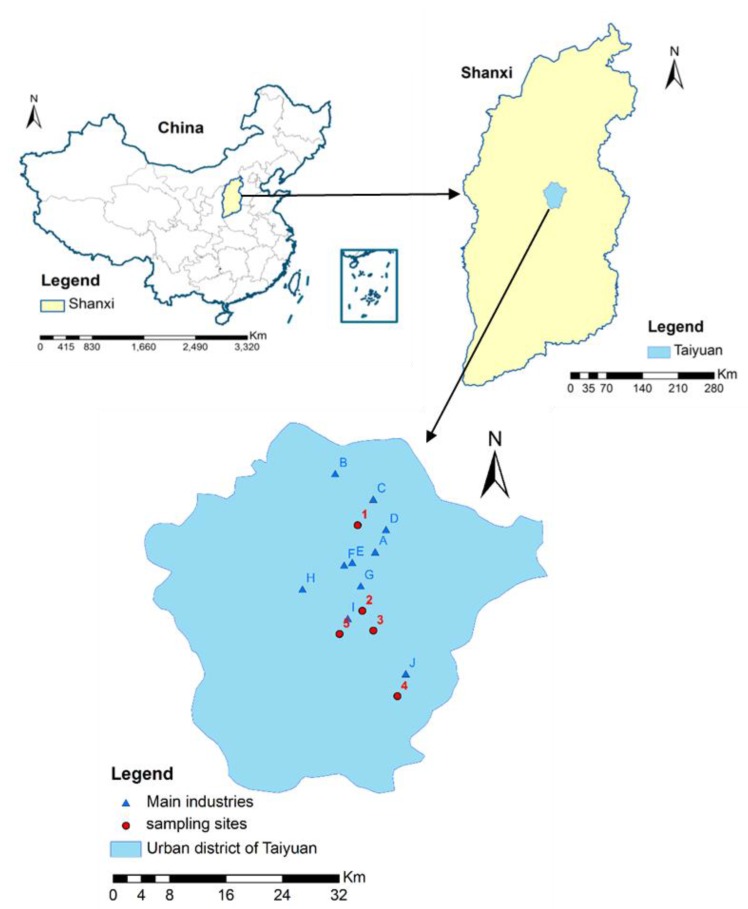
Sampling sites of wastewater treatment plants (WWPTs) and main industries located in the urban district of Taiyuan, China (A is iron and steel industry; B is metallurgical industry; C and I are chemical industry; D, E and F are engineering machinery industry; G is heavy industry; H is electricity industry and J is FOXCONN).

To evaluate the human health risk of exposure to heavy metals in sewage sludge, samples were collected from 5 WWTPs in the urban district of Taiyuan—the capital of Shanxi—as indicated in Figure 1. The location and treatment technologies of different WWTPs are shown in Table 1. Four subsamples were collected from different sites in the same WWTP, and then the samples were mixed into a single sample to ensure accuracy of representation. 

### 2.2. Determination of the Total Heavy Metal Concentration 

In the laboratory, sewage sludge samples were air-dried at room temperature for one week, sieved through a mesh with a 0.14 mm pore size, sealed in brown glass bottles, and stored at room temperature [25]. The samples were weighted (0.200 g) and digested with HNO_3_ using a microwave digestion system (Mars 5, CEM, Saint Matthews, NC, USA) according to the USEPA Method 3051B [26]. The temperature of each sample was raised to 175 °C in less than 5.5 min and held between 170 and 180 °C for the balance of the 10-minute irradiation period [26]. This strong acid digestion method dissolves almost all of the elements that could become environmentally available. After that, all sample solutions were filtered through filter paper, quantitatively transferred to a volumetric flask and then diluted with deionized water to a total volume of 50 mL. Next, Cu, Zn, Pb and Cr were analyzed using an atomic absorption spectrophotometer (WARIAN-AA-240, WARIAN, Palo Alto, CA, USA), As and Hg were analyzed using an atomic fluorescence spectrometer (AFS-230E, Haiguang, Beijing, China), and Cd was analyzed using a graphite furnace atomic absorption spectrophotometer (TAS-990AFG, PERSEE, Beijing, China). Each batch of samples was tested simultaneously with blank samples and reference samples. Samples were measured in triplicate, and the mean values of the results were reported as the final concentration of the heavy metals. To control the quality, standard reference sludge samples (RTC-CRM055, TMRM, Shanghai, China) and national standards of China (GB/T 15555.2-1995, GB/T 15555.2-1995, GB/T 15555.2-1995, GB/T 15555.6-1995, GB/T 22105.2-2008, GB/T 22105.1-2008 and GB/T 17141-1997) were used. To ensure the accuracy and precision of the measurements, the certified sewage sludge was tested to confirm the accuracy, precision and recovery. Blank samples were also tested 11 times to check the method detection limits. The analytical accuracy, precision, recovery and method detection limit were showed in the Table 2 as follows.

### 2.3. Health Risk Assessment 

The human health risk assessment is used to estimate the potential health risk and the probability of adverse human health effects caused by chemicals in a contaminated environment [27,28,29]. Three steps are performed to assess the human health risk of heavy metals: hazard identification, exposure assessment and risk assessment [30].

Based on the USEPA Part 503 rule, Cu, Zn, As, Hg, Pb, Cr and Cd were identified as toxic heavy metals that have adverse effects on human health, and ingestion and inhalation are the two main pathways of human exposure to heavy metals in sewage sludge [19]. Furthermore, there are two forms that can be inhaled by humans: volatilized sewage sludge and particles (dust) [19]. Cu, Zn, Hg, Pb, and Cr are classified as non-carcinogenic pollutants by the USEPA (USEPA 2002) [25].As and Cd are classified as carcinogenic pollutants by the International Agency for Research on Cancer (IARC) and the World Health Organization (WHO) [31,32]. 

#### 2.3.1. Exposure Assessment

To evaluate the human health risk of exposure to heavy metals in sewage sludge via ingestion and inhalation, the average daily dose (ADD) (mg·kg^−1^·day^−1^) of heavy metals was determined using the following equations [20,21,25,30,31,33]:

$${\mathrm{ADD}}_{\mathrm{ingest}}=\frac{C\times {\mathrm{IR}}_{\mathrm{ingest}}\times \mathrm{EF}\times \mathrm{ED}}{\mathrm{BW}\times \mathrm{AT}}\times \mathrm{CF}$$

$${\mathrm{ADD}}_{\mathrm{inhale}}=\frac{C\times \mathrm{InhR}\times \mathrm{EF}\times \mathrm{ED}}{\mathrm{PEF}\times \mathrm{BW}\times \mathrm{AT}}$$

where ${\mathrm{ADD}}_{\mathrm{ingest}}$ is the average daily dose for ingestion, mg·kg^−1^·day^−1^; ${\mathrm{ADD}}_{\mathrm{inhale}}$ is the average daily dose for inhalation, mg·kg^−1^·day^−1^; C is the concentration of heavy metals in sewage sludge, mg·kg^−1^, which was the pollutant concentration in the environment but not the concentration that humans were exposed to via ingestion or inhalation; ${\mathrm{IR}}_{\mathrm{ingest}}$ is the ingestion rate of heavy metals, mg·day^−1^, and is 100 mg·day^−1^ for adults and 200 mg·day^−1^ for children [34]; EF is the exposure frequency, days·year^−1^, with 350 days·year^−1^ [34]; ED is the exposure duration, years, and is 30 years for adults and 6 years for children; BW is the average body weight, kg, which is 70 kg for adults and 16 kg for children [19]; AT is the averaging time, days, and this value for non-carcinogens is equal to ED × 365 days and for carcinogens is equal to 70 years (lifetime) × 365 days [34]; CF is a conversion factor, 1 × 10^−6^, and is a unity conversion factor; InhR is the inhalation rate, m^3^·day^−1^, which is 7.6 m^3^·day^−1^ for children and 20 m^3^·day^−1^ for adults [34]; and PEF is the particle emission factor, 1.36 × 10^9^ m^3^·kg^−1^, which is the sewage sludge-to-air particulate emission factor [30].

#### 2.3.2. Non-Carcinogenic Risk Assessment 

The hazard quotient (HQ) was applied to assess the non-carcinogenic risk, which was defined as the ratio of the average daily dose of each heavy metal via each exposure pathway and the reference dose (RfD). The equation to calculate this index is as follows [20,31,35,36]:

$${\mathrm{HQ}}_{\mathrm{ij}}=\frac{{\mathrm{ADD}}_{\mathrm{ij}}}{{\mathrm{RfD}}_{\mathrm{ij}}}$$

where HQ_ij_ is the hazard quotient of the ith heavy metal via the jth pathway; ADD_ij_ is the average daily dose for the ith heavy metal via the jth pathway, mg·kg^−1^·day^−1^; and RfD_ij_ is the risk reference dose of the ith heavy metal via the jth pathway, mg·kg^−1^·day^−1^, which is the maximum allowable content of heavy metal via different pathways that pose no harmful effects on human health. The value of each heavy metal and As via different pathways is listed in Table 3. In this study, the RfD via ingestion and inhalation was considered.

To assess the overall non-carcinogenic effects of exposure to multiple heavy metals via different pathways, the sum of the HQ values of all heavy metals via all pathways is expressed as the hazard index (HI). The equation to calculate this index is as follows [37]:

$$\mathrm{HI}=\overset{n}{\underset{i=1}{\sum }}\overset{m}{\underset{j=1}{\sum }}{\mathrm{HQ}}_{\mathrm{ij}}$$

If the values of HQ and HI are less than 1, then humans are unlikely to experience obvious adverse health effects; if the HQ and HI values are more than 1, then humans may tolerate the non-carcinogenic risk, and there is a chance that non-carcinogenic effects may occur with a probability that tends to increase as HQ and HI increase [20,21,25,38].

#### 2.3.3. Carcinogenic Risk Assessment

The carcinogenic risk is the probability of an individual developing cancer over a lifetime as a result of exposure to a potential carcinogen, and it is expressed by the index RISK [30]. If there are multiple carcinogenic contaminations, the cancer risk of all carcinogens and exposure pathways are summed. The equation to calculate this index is as follows [20,21,31]:

RISK=ADD×SF

where SF is the carcinogenic slope factor, mg·day·mg^−1^, which is used to quantify the human cancer risk from sewage sludge and represents the dose at which an exposed individual would be expected to get cancer [19]. The values of SF are shown in Table 3.

For a single heavy metal, a RISK of less than 1 × 10^−6^ is regarded as inconsequential and the cancer risk can be ignored; While a RISK of more than 1 × 10^−4^ is regarded as unacceptable and the cancer risk is concerning. For the sum of all heavy metals via all exposure pathways, the acceptable level is 1 × 10^−5^ [20,32,37].

Studies have shown that incidence of cancer of the skin, bladder, lung and respiratory system increases significantly when humans are exposed to As, and exposure to a high concentration of Cd can cause cancer that primarily affects the lung and prostate [39,40,41].

## 3. Results and Discussion

### 3.1. Heavy Metals and As Concentrations in Sewage Sludge from Different WWTPs in Taiyuan

The heavy metals and As concentrations in sewage sludge from five different WWTPs in Taiyuan are presented in Table 4. The mean concentrations of heavy metals and As can be ranked in the following decreasing order: Cu > Cr > Zn > Pb > As > Hg > Cd. Cu was the most abundant metal in sewage sludge, and Cd was the least abundant metal. The mean concentration of Cu, Cr, Zn, Pb, As, Hg and Cd was 214.08 mg/kg, 111.54 mg/kg, 93.64 mg/kg, 50.84 mg/kg, 16.69 mg/kg, 2.80 mg/kg and 0.68 mg/kg respectively. There was a significant variation in the heavy metals and As concentrations in the sewage sludge samples because the samples were collected from different WWTPs. The metals and As can be ranked by standard deviation in the following decreasing order: Cu > Cr > Zn > Pb > As > Hg > Cd. There was an obvious change in the concentration of Cu, Cr and Zn. 

As shown in Table 5, the limit values of the seven heavy metals and As established by different countries are generally the same, except in Canada where the limit of As is less than the value established by other countries [14]. Compared with the threshold values, the contents of heavy metals and As in sewage sludge samples were lower, except for the As content, which was higher than the limit value established by Canada. This result showed that, with the exception of As, the heavy metals contents in sewage sludge from the study area were not high. The high As values can be attributed to the coal and coking industries are were distributed in Taiyuan.

### 3.2. Exposure Assessment

The daily exposure to heavy metals and As in sewage sludge was determined according to the methods discussed previously. The mean and 95% upper confidence limit (UCL) exposures of heavy metals and As for adults and children via ingestion and inhalation are shown in Table 6.

Based on the mean and 95% UCL values of the ADD, heavy metals and As can be ranked in decreasing order for adults as Cu > Cr > Zn > Pb > As > Hg > Cd and for children as Cu > Cr > Zn > Pb > Hg > As > Cd. According to both the mean and 95% UCL, the highest ADD values for all adults and children were recorded for Cu, Cr and Zn, followed by Pb, As and Hg, and the lowest value measured was for Cd. For children, the exposure to all heavy metals, except Cd, was an order of magnitude higher than that for adults, and the exposure to Cd was also slightly higher for children than for adults. Furthermore, the total heavy metals exposure for children was 8.65 and 9.93 times higher than that for adults in the mean value and 95% UCL. As in other studies, this result indicated that heavy metals exposure based on the same concentration was higher for children than for adults, and the harmful effects of heavy metals and As exposure in sewage sludge was more serious for children [31,42]. This finding may be because the body weight of children is less than that of adults, and compared with adults, children participate in outdoor activities more often [32,42].

Comparing the different pathways of heavy metals and As exposure, ingestion was the main pathway for heavy metals and As exposure, and inhalation played a very small role for both adults and children. This is consistent with findings of other researchers [37,43,44]. 

### 3.3. Health Risk Assessment

#### 3.3.1. Non-Carcinogenic Health Risk

The non-carcinogenic health risks due to exposure to heavy metals in sewage sludge via ingestion and inhalation are shown in Table 7. Due to the high concentration of Cu and Cr and the low RfD values of Hg, Cu, Hg and Cr, these metals showed a higher non-carcinogenic risk for both adults and children than Zn and Pb. 

According to the mean value of HQ, Cu, Hg and Cr accounted for 50.74%, 26.51% and 21.18% of the HI value, respectively, for adults, and these values were 50.80%, 26.51% and 21.13% of the HI value, respectively, for children. According to the 95% UCL, the ratio was 47.64%, 27.16% and 23.85% for adults and 44.89%, 26.98% and 27.24% for children. By contrast, the total percentage of Zn and Pb for HI was only 1.57% and was the same value for adults and for children according to the mean value of HQ. Broken down, the values for Zn and Pb were 0.27% and 1.07% for adults, 0.17% and 0.96% for children according to the 95% UCL. Based on the mean and 95% UCL, heavy metals can be ranked in the same decreasing order by their HQ values as Cu > Hg > Cr > Pb > Zn for both adults and children. 

The values of HQ_ingest_ and HQ_inhale_ were less than 1 for all heavy metals, except for the HQ_ingest_ of Cu for children in the 95% UCL which was 1.01. This indicated that there was no non-carcinogenic risk when adults and children were exposed to one type of heavy metal in sewage sludge via ingestion or inhalation, except that children had a non-carcinogenic risk of Cu in the 95% UCL via ingestion. The large difference between HQ_ingest_ and HQ_inhale_ indicated that ingestion was the main pathway of heavy metal exposure in sewage sludge for adults and children, and the inhalation pathways could be neglected.

Accordingly, based on the mean value, the calculated HQ was less than 1 for both adults and children, and the HQ for the 95% UCL for Cu was more than 1 for children. This result suggested that in the mean value, neither adults nor children would suffer a potential health risk when exposed to only one type of heavy metal. However, in the 95% UCL, children suffered a potential non-carcinogenic risk. In addition, compared to the value for adults, the HQ for children was higher for the mean and 95% UCL, which indicated that the capacity of children to respond to pollutants was weaker than that of adults when they were exposed to the same toxin [43,45]. 

The HI values for adults were 0.144 and 0.208 for the mean and 95% UCL, which is less than 1, and for children, these values were 1.26 and 2.25, which is higher than 1. This result implied that children had non-carcinogenic risk and that heavy metals in sewage sludge would be toxic to children but not to adults; thus, children were more vulnerable to heavy metals than adults [16,23].

**Figure 2:**
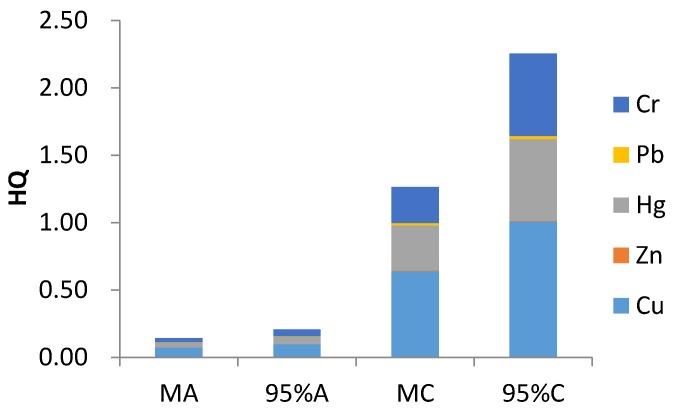
The HQ for non-carcinogenic risks for humans (adults and children) based on the mean and 95% UCL contents (MA, 95%A, MC and 95%C represented Mean for adults, 95% upper confidence limit (UCL) for adults, mean for children and 95% UCL for children).

As shown in Figure 2, the results indicated that Cu was the major source of non-carcinogenic risk in sewage sludge, and ingestion was the primary pathway for non-carcinogenic risk [17,28,46]. Compared with adults, children were found to be more susceptible to the non-carcinogenic risk induced by heavy metals [47].

#### 3.3.2. Carcinogenic Health Risk

RISK is assessed by calculating the incremental probability of an individual developing cancer over a life time as a result of exposure to a potential carcinogen. To assess the carcinogenic risk, the risk exposure to Cd and As via ingestion and inhalation were calculated. Results are shown in Table 8. According to the mean value and 95% UCL, the RISK_ingest_ and RISK_inhale_ values for As and Cd were less than the safe limit of 1 × 10^−4^, revealing that there was no carcinogenic risk when both adults and children were exposed to only As or Cd. 

Furthermore, as shown in Table 8, according to the mean value, the total carcinogenic risk values of RISK were 1.71 × 10^−5^ for adults and 3.00 × 10^−5^ for children, respectively; by the 95% UCL, the total carcinogenic risk values of RISK were 2.29 × 10^−5^ for adults and 3.51 × 10^−5^ for children. Regardless of whether the mean value or the 95% UCL was used, the RISK was higher for children than for adults, and all values exceeded the limit of 1 × 10^−5^. This result indicated that both adults and children suffered a carcinogenic risk, and children suffered from more carcinogenic risk than adults. Comparing the RISK values, the value for As was larger than for Cd, implying that As was the main pollutant for carcinogenic risk. Moreover, the RISK_ingest_ values were all larger than the RISK_inhale_ value, which implied that ingestion was the main pathway for carcinogenic risk.

**Figure 3:**
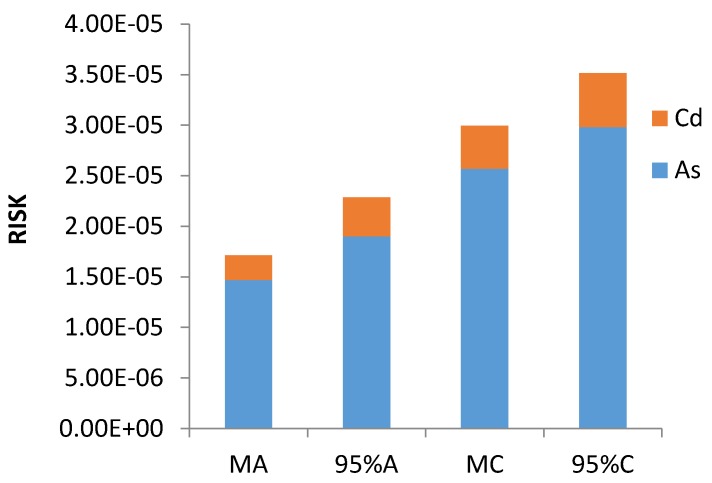
The RISK for carcinogenic risks for humans (adults and children) based on the mean and 95% UCL contents.

As shown in Figure 3, the results indicated that both adults and children had carcinogenic risk. Moreover, As posed a larger carcinogenic risk than Cd in sewage sludge, and the carcinogenic risk primarily arose via the ingestion pathway [25]. Comparing the results of RISK for adults and children, children suffered from more carcinogenic risk than adults, implying that children were more sensitive and vulnerable to heavy metals in sewage sludge [23,43,48].

### 3.4. Uncertainty Analysis 

Uncertainty analysis of human health risk for adults and children was carried out according to the Monte Carlo model. The results of the uncertainty analysis indicated that the range of non-carcinogenic risk for adults was 5.01 × 10^−2^–2.43 × 10^−1^, for children was 4.62 × 10^−1^–2.12 × 10^0^; and the range of carcinogenic risk for adults was 1.72 × 10^−5^–2.77 × 10^−5^, for children was 1.26 × 10^−5^–4.85 × 10^−5^. Based on the uncertainty analysis, the non-carcinogenic risk and carcinogenic risk for children were all greater than that for adults, which is consistent with the preceding certainty analysis. Moreover, the range of health risk based on uncertainty analysis was greater than what was found with the certainty analysis. This implied that uncertainty analysis was more conservative. 

## 4. Conclusions

To compare the health risk of heavy metals and As in sewage sludge from WWTPs between adults and children, samples were collected from five WWPTs located in Taiyuan, the capital of Shanxi. The contents of heavy metals and As in the sewage sludge were determined, and they were ranked in the following order using the mean values: Cu > Cr > Zn > Pb > As > Hg > Cd. Compared with the concentration limits set by different countries, all heavy metals in sewage sludge were within the standard limits, except that the content of As was higher than the limit value established by Canada. According to the mean and 95% UCL content of heavy metals and As, ingestion was the main pathway for heavy metals and As exposure, and inhalation played a very small role. For total exposure, the ADD value for children was 8.65 and 9.93 times higher than the ADD value for adults for the mean and 95% UCL contents, respectively. Regarding non-carcinogenic risk, heavy metals can be ranked according to the HQ values as Cu > Hg > Cr > Pb > Zn for both adults and children. Cu was the main source and ingestion was the main pathway for non-carcinogenic risk for both adults and children. Furthermore, the HI was more than the limit value of 1 for children but less than the limit for adults. The results of RISK indicated that As was the main source and ingestion was the main pathway for carcinogenic risk, and both adults and children suffered from this risk. Based on the uncertainty analysis, the range of non-carcinogenic risk for adults was 5.01 × 10^−2^–2.43 × 10^−1^, for children was 4.62 × 10^−1^–2.12 × 10^0^; and the range of carcinogenic risk for adults was 1.72 × 10^−5^–2.77 × 10^−5^, for children was 1.26 × 10^−5^–4.85 × 10^−5^. It implied that the non-carcinogenic risk and carcinogenic risk for children were all greater than that for adults and uncertainty analysis was more conservative than certainty analysis. 

By comparing the health risk to adults and children of heavy metals and As in sewage sludge, regardless of the carcinogenic or non-carcinogenic risk, children were more susceptible to the potential health risk due to the presence of the heavy metals and As in sewage sludge. All of the results implied that in the same adverse environment, children are more sensitive and vulnerable than adults; thus, more attention should be given to children to avoid the harmful effects of pollutants. 

## Figures and Tables

**Table 1:** The location and treatment technologies of different WWTPs in the urban district of Taiyuan.

No.	Name	Latitude	Longitude	Treatment Technology
1	Taiyuan drainage management office sewage purification II plant	112.530767	37.940478	Carrousel oxidation ditch
2	Taiyuan Xinlangming Sewage Treatment Co., Ltd.	112.536829	37.831682	Anaerobic/Anoxic/Oxic
3	Taiyuan Haofeng Sewage Treatment Co., Ltd.	112.550556	37.806389	Activated sludge process
4	Taiyuan South Weir Sewage Treatment Branch	112.581518	37.722606	Biological contact oxidation process
5	Taiyuan Golden Century Sunshine Water Purification Co., Ltd.	112.507611	37.802056	Activated sludge process

**Table 2:** Analytical accuracy, precision, recovery and method detection limit.

Heavy Metal	Certified Value (mg/kg)	Measured Value (mg/kg)	Accuracy (%)	Precision (%)	Recovery (%)	Method Detection Limit (mg/kg)
Cu	482	471.04	4.31	−2.27	94.40%	1.03
Zn	1240	1229.91	5.77	−0.81	93.00%	0.97
Hg	12.5	11.40	0.76	−8.81	91.20%	0.0042
Pb	154	147.89	3.25	−3.97	92.95%	0.28
Cr	289	281.41	4.60	−2.63	94.80%	4.83
As	229	221.91	3.88	−3.09	95.75%	0.015
Cd	60	62.92	1.30	4.87	105.55%	0.0056

**Table 3:** Reference dose (RfD) and carcinogenic slope factor (SF) for different heavy metals ^a,b^.

Heavy Metal	RfD (mg·kg^−1^·d^−1^)		SF (kg·d·mg^−1^)
	Ingestion	Inhalation	
Cu	0.004	0.004	
Zn	0.300	0.300	
Hg	0.0001	0.0001	
Pb	0.038	0.038	
Cr	0.005	0.005	
As			1.5
Cd			6.1

^a^ USEPA. Risk assessment guidance for superfund, volume I, human health evaluation manual (Part A), 1989; ^b^ USEPA. Risk Assessment Information System database.

**Table 4:** Heavy metals concentrations in sewage sludge from WWTPs in the urban district of Taiyuan (mg·kg^−1^).

Heavy Metal	MIN	MAX	Mean	Standard Deviation
Cu	149.94	261.00	214.08	54.30
Zn	63.44	121.10	93.64	21.86
Hg	1.72	3.74	2.80	0.96
Pb	41.13	57.39	50.84	7.82
Cr	54.48	186.24	111.54	49.91
As	13.85	22.51	16.69	3.56
Cd	0.33	1.06	0.68	0.29

**Table 5:** Heavy metals and As limit values in sewage sludge for agricultural use (mg·kg^−1^).

	Cu	Zn	Hg	Pb	Cr	As	Cd
USEPA ^a^	1500	2800	-	300	1200	41	39
European Union ^b^	1000–1750	2500–4000	16–25	750–1200	-	-	20–40
Spain ^c^							
pH < 7	1000	2500	16	750	300	-	20
pH > 7	1750	4000	25	1200	400	-	40
Canada ^d^	500	2000	10	200	1000	10	20
CJ/T 309-2009 ^e^							
Grade A ^f^	500	1500	3	300	500	30	3
Grade B ^g^	1500	3000	15	1000	1000	75	15

^a^ USEPA. A guide to the biosolids risk assessments for the EPA Part 503 rule, 1995; ^b^ EUR-lex. Council directive on the protection of the environment, and in particular of the soil, when sewage sludge is used in agriculture, 1986; ^c^ Przewrocki, P.; Kulczycka, J.; Wzorek, Z.; Kowalski, Z.; Gorazda, K.; Jodko, M. Risk analysis of sewage sludge—Poland and EU comparative approach. *Polish Journal of Environmental Studies* 2004, 13, 39–59; ^d^ Cao, J.Z. The agricultural value analysis of surplus sludge from municipal wastewater treatment plant. Taiyuan SCI-TECH 2003, 3, 14–15; ^e^ Disposal of sludges from municipal wastewater treatment plant—Control standard for agricultural use (CJ/T 309-2009); ^f^ Grade A applies to vegetables and food crops; ^g^ Grade B applies to oil crops, fruit trees, feed crops, and fiber crops.

**Table 6:** The average daily dose (ADD) of heavy metals in sewage sludge from WWPTs in the urban district of Taiyuan (mg·kg^−1^·day^−1^).

Heavy Metal		Adults			Children		
		ADD_ingest_	ADD_inhale_	ADD	ADD_ingest_	ADD_inhale_	ADD
Cu	Range	2.05 × 10^−4^–3.58 × 10^−4^	3.02 × 10^−8^–5.26 × 10^−8^	2.05 × 10^−4^–3.58 × 10^−4^	1.80 × 10^−3^–3.13 × 10^−3^	5.02 × 10^−8^–8.74 × 10^−8^	1.80 × 10^−3^–3.13 × 10^−3^
	Mean	2.93 × 10^−4^	4.31 × 10^−8^	2.93 × 10^−4^	2.57 × 10^−3^	7.17 × 10^−8^	2.57 × 10^−3^
	95% UCL	3.97 × 10^−4^	6.78 × 10^−8^	3.97 × 10^−4^	4.03 × 10^−3^	1.13 × 10^−7^	4.03 × 10^−3^
Zn	Range	8.69 × 10^−5^–1.66 × 10^−4^	1.28 × 10^−8^–2.44 × 10^−8^	8.69 × 10^−5^–1.19 × 10^−4^	7.60 × 10^−4^–1.45 × 10^−3^	2.12 × 10^−8^–4.06 × 10^−8^	7.60 × 10^−4^–1.04 × 10^−3^
	Mean	1.28 × 10^−4^	1.89 × 10^−8^	1.28 × 10^−4^	1.12 × 10^−3^	3.14 × 10^−8^	1.12 × 10^−3^
	95% UCL	1.70 × 10^−4^	1.89 × 10^−8^	1.70 × 10^−4^	1.12 × 10^−3^	3.14 × 10^−8^	1.12 × 10^−3^
Hg	Range	2.36 × 10^−6^–5.12 × 10^−6^	3.46 × 10^−10^–7.53 × 10^−10^	2.36 × 10^−6^–5.12 × 10^−6^	2.06 × 10^−5^–4.48 × 10^−5^	5.76 × 10^−10^–1.25 × 10^−9^	2.06 × 10^−5^–4.48 × 10^−5^
	Mean	3.83 × 10^−6^	5.64 × 10^−10^	3.83 × 10^−6^	3.35 × 10^−5^	9.37 × 10^−10^	3.35 × 10^−5^
	95% UCL	5.65 × 10^−6^	1.02 × 10^−9^	5.65 × 10^−6^	6.07 × 10^−5^	1.70 × 10^−9^	6.07 × 10^−5^
Pb	Range	5.63 × 10^−5^–7.86 × 10^−5^	8.29 × 10^−9^–1.16 × 10^−8^	5.64 × 10^−5^–7.86 × 10^−5^	4.93 × 10^−4^–6.88 × 10^−4^	1.38 × 10^−8^–1.92 × 10^−8^	4.93 × 10^−4^–6.88 × 10^−4^
	Mean	6.96 × 10^−5^	1.02 × 10^−8^	6.96 × 10^−5^	6.09 × 10^−4^	1.70 × 10^−8^	6.09 × 10^−4^
	95% UCL	8.45 × 10^−5^	1.37 × 10^−8^	8.45 × 10^−5^	8.18 × 10^−4^	2.29 × 10^−8^	8.18 × 10^−4^
Cr	Range	2.55 × 10^−4^–7.46 × 10^−5^	1.10 × 10^−8^–3.75 × 10^−8^	7.46 × 10^−5^–2.55 × 10^−4^	6.53 × 10^−4^–2.23 × 10^−3^	1.82 × 10^−8^–6.24 × 10^−8^	6.53 × 10^−4^–2.23 × 10^−3^
	Mean	1.53 × 10^−4^	2.25 × 10^−8^	1.53 × 10^−4^	1.34 × 10^−3^	3.74 × 10^−8^	1.34 × 10^−3^
	95% UCL	2.48 × 10^−4^	5.15 × 10^−8^	2.48 × 10^−4^	3.06 × 10^−3^	8.56 × 10^−8^	3.06 × 10^−3^
As	Range	8.13 × 10^−6^–1.32 × 10^−5^	1.20 × 10^−9^–1.94 × 10^−9^	8.13 × 10^−6^–1.03 × 10^−5^	1.42 × 10^−5^–2.31 × 10^−5^	3.97 × 10^−10^–6.46 × 10^−10^	1.42 × 10^−5^–2.31 × 10^−5^
	Mean	9.80 × 10^−6^	1.44 × 10^−9^	9.80 × 10^−6^	1.71 × 10^−5^	4.79 × 10^−10^	1.71 × 10^−5^
	95% UCL	1.27 × 10^−5^	1.67 × 10^−9^	1.27 × 10^−5^	1.99 × 10^−5^	5.55 × 10^−10^	1.99 × 10^−5^
Cd	Range	1.95 × 10^−7^–6.21 × 10^−7^	2.87 × 10^−11^–9.14 × 10^−11^	1.95 × 10^−7^–6.21 × 10^−7^	3.42 × 10^−7^–8.25 × 10^−7^	3.04 × 10^−11^–9.55 × 10^−12^	3.42 × 10^−7^–1.09 × 10^−6^
	Mean	3.98 × 10^−7^	5.85 × 10^−11^	3.98 × 10^−7^	6.96 × 10^−7^	1.94 × 10^−11^	6.96 × 10^−7^
	95% UCL	6.33 × 10^−7^	7.36 × 10^−11^	6.34 × 10^−7^	8.76 × 10^−7^	2.45 × 10^−11^	8.76 × 10^−7^
Sum	Mean	6.58 × 10^−4^	9.68 × 10^−8^	6.58 × 10^−4^	5.69 × 10^−3^	1.59 × 10^−7^	5.69 × 10^−3^
	95% UCL	9.18 × 10^−4^	1.55 × 10^−7^	9.18 × 10^−4^	9.12 × 10^−3^	2.55 × 10^−7^	9.12 × 10^−3^

**Table 7:** Non-carcinogenic risks for humans (adults and children) due to environmental exposure to heavy metals of hazard quotient (HQ) in sewage sludge from WWPTs in the urban district of Taiyuan.

Heavy Metal		Adults			Children		
		HQ_ingest_	HQ_inhale_	HQ	HQ_ingest_	HQ_inhale_	HQ
Cu	Range	5.13 × 10^−2^–8.94 × 10^−2^	7.55 × 10^−6^–1.31 × 10^−5^	5.14 × 10^−2^–8.94 × 10^−2^	4.49 × 10^−1^–7.82 × 10^−1^	1.26 × 10^−5^–2.19 × 10^−5^	4.49 × 10^−1^–7.82 × 10^−1^
	Mean	7.33 × 10^−2^	1.08 × 10^−5^	7.33 × 10^−2^	6.42 × 10^−1^	1.79 × 10^−5^	6.42 × 10^−1^
	95% UCL	9.91 × 10^−2^	1.69 × 10^−5^	9.91 × 10^−2^	1.01 × 10^0^	2.82 × 10^−5^	1.01 × 10^0^
Zn	Range	2.90 × 10^−4^–5.53 × 10^−4^	4.26 × 10^−8^–8.13 × 10^−8^	2.90 × 10^−4^–5.53 × 10^−4^	2.53 × 10^−3^–4.84 × 10^−3^	7.08 × 10^−8^–1.35 × 10^−7^	2.53 × 10^−3^–4.28 × 10^−3^
	Mean	4.28 × 10^−4^	6.29 × 10^−8^	4.28 × 10^−4^	3.74 × 10^−3^	1.05 × 10^−7^	3.74 × 10^−3^
	95% UCL	5.66 × 10^−4^	6.30 × 10^−8^	5.66 × 10^−4^	3.75 × 10^−3^	1.05 × 10^−7^	3.75 × 10^−3^
Hg	Range	2.36 × 10^−2^–5.12 × 10^−2^	3.46 × 10^−6^–7.53 × 10^−6^	2.36 × 10^−2^–5.12 × 10^−2^	2.06 × 10^−1^–4.48 × 10^−1^	1.25 × 10^−5^–5.76 × 10^−6^	2.06 × 10^−1^–4.48 × 10^−1^
	Mean	3.83 × 10^−2^	5.64 × 10^−6^	3.83 × 10^−2^	3.35 × 10^−1^	9.37 × 10^−6^	3.35 × 10^−1^
	95% UCL	5.65 × 10^−2^	1.02 × 10^−5^	5.65 × 10^−2^	6.07 × 10^−1^	1.70 × 10^−5^	6.07 × 10^−1^
Pb	Range	1.48 × 10^−3^–2.07 × 10^−3^	2.18 × 10^−7^–3.04 × 10^−7^	1.48 × 10^−3^–2.07 × 10^−3^	1.30 × 10^−2^–1.81 × 10^−2^	3.63 × 10^−7^–5.06 × 10^−7^	1.30 × 10^−2^–1.81 × 10^−2^
	Mean	1.83 × 10^−3^	2.69 × 10^−7^	1.83 × 10^−3^	1.60 × 10^−2^	4.48 × 10^−7^	1.60 × 10^−2^
	95% UCL	2.22 × 10^−3^	3.62 × 10^−7^	2.22 × 10^−3^	2.15 × 10^−2^	6.02 × 10^−7^	2.15 × 10^−2^
Cr	Range	1.49 × 10^−2^–5.10 × 10^−2^	2.19 × 10^−6^–7.50 × 10^−6^	1.49 × 10^−2^–5.10 × 10^−2^	1.31 × 10^−1^–4.46 × 10^−1^	3.65 × 10^−6^–1.25 × 10^−5^	1.31 × 10^−1^–4.46 × 10^−1^
	Mean	3.06 × 10^−2^	4.49 × 10^−6^	3.06 × 10^−2^	2.67 × 10^−1^	7.47 × 10^−6^	2.67 × 10^−1^
	95% UCL	4.95 × 10^−2^	1.03 × 10^−5^	4.96 × 10^−2^	6.13 × 10^−1^	1.71 × 10^−5^	6.13 × 10^−1^
HI	Range	9.89 × 10^−2^–1.94 × 10^−1^			8.65 × 10^−1^–1.26 × 10^0^		
	Mean	1.44 × 10^−1^			1.26 × 10^0^		
	95% UCL	2.08 × 10^−1^			2.25 × 10^0^		

**Table 8:** Carcinogenic risks for humans (adults and children) due to environmental exposure to heavy metals in sewage sludge from WWPTs in the urban district of Taiyuan.

Heavy Metal		Adults			Children		
		RISK_ingest_	RISK_inhale_	RISK	RISK_ingest_	RISK_inhale_	RISK
As	Range	1.22 × 10^−5^–1.98 × 10^−5^	1.79 × 10^−9^–2.91 × 10^−9^	1.22 × 10^−5^–1.98 × 10^−5^	2.13 × 10^−5^–3.47 × 10^−5^	5.96 × 10^−10^–9.69 × 10^−10^	2.13 × 10^−5^–3.47 × 10^−5^
	Mean	1.47 × 10^−5^	2.16 × 10^−9^	1.47 × 10^−5^	2.57 × 10^−5^	7.19 × 10^−10^	2.57 × 10^−5^
	95% UCL	1.90 × 10^−5^	2.50 × 10^−9^	1.90 × 10^−5^	2.98 × 10^−5^	8.32 × 10^−10^	2.98 × 10^−5^
Cd	Range	1.19 × 10^−6^–3.79 × 10^−6^	1.75 × 10^−10^–5.57 × 10^−10^	1.19 × 10^−6^–3.79 × 10^−6^	2.08 × 10^−6^–6.63 × 10^−6^	5.82 × 10^−11^–1.85 × 10^−10^	2.08 × 10^−6^–6.63 × 10^−6^
	Mean	2.43 × 10^−6^	3.57 × 10^−10^	2.43 × 10^−6^	4.24 × 10^−6^	1.19 × 10^−10^	4.24 × 10^−6^
	95% UCL	3.86 × 10^−6^	4.49 × 10^−10^	3.86 × 10^−6^	5.34 × 10^−6^	1.49 × 10^−10^	5.34 × 10^−6^
RISK	Range	1.43 × 10^−5^–2.10 × 10^−5^			2.50 × 10^−5^–3.68 × 10^−5^		
	Mean	1.71 × 10^−5^			3.00 × 10^−5^		
	95% UCL	2.29 × 10^−5^			3.51 × 10^−5^		
